# ‘Alternative’ cancer cures via the Internet?

**DOI:** 10.1038/sj.bjc.6600513

**Published:** 2002-08-27

**Authors:** E Ernst, K Schmidt

**Affiliations:** Department of Complementary Medicine, School of Postgraduate Medicine and Health Sciences, University of Exeter, 25 Victoria Park Road, Exeter EX2 4NT, UK

## Abstract

*British Journal of Cancer* (2002) **87**, 479–480. doi:10.1038/sj.bjc.6600513 www.bjcancer.com

© 2002 Cancer Research UK

## 

‘Alternative’ cancer cures (ACCs) continue to be heavily promoted (Ernst *et al*, 1999). Conventional oncologists often have limited knowledge about ACCs (Newell *et al*, 2000), and compelling evidence is usually not available (Ernst *et al*, 2001). Cancer patients tend to get confused in the maze of claims and counter-claims and often turn to the ‘Internet’ for information. Following advice obtainable via the ‘world wide web’ can, however, be hazardous. Tragic instances where this brought considerable harm, even death, to cancer patients are on record (e.g. Hainer *et al*, 2000). It is therefore reasonable to ask whether the advice offered to cancer patients by some of the most prominent ‘web sites’ might put cancer patients at risk.

We identified eight popular search engines (Mansoor, 2001; Sullivan and Nielsen (www.searchenginewatch.com)), and searched for the following terms between 18th and 25th September, 2001: ‘complementary medicine’ or ‘alternative medicine’ or ‘complementary therapy’ or ‘alternative therapy’ and ‘cancer’. The first 30 hits from each search engine were compared. All ‘web sites’ which were listed on at least three search engines were evaluated and rated on a scale of 0–14 (Sandvik, 1999).

Table 1

**Table 1:**
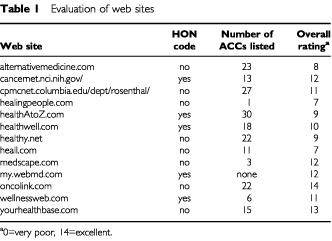
Evaluation of web sites

summarises our evaluation of the 13 ‘sites’ that could be included in our analysis. Three of the ‘sites’ overtly discouraged cancer patients to employ conventional therapies. Most ‘sites’ recommended a multitude of treatments with little consensus between them. Cancer prevention was advocated on all ‘sites’. In our judgement, five ‘sites’ had the potential to harm cancer patients if the advice provided was followed. Our overall rating for the ‘sites’ ranged from medium to good (Table 1).

These findings suggest that the quality of the information of the ‘web sites’ offered to cancer patients is highly variable. The vast majority recommends ACCs for which there is no evidence of efficacy (Ernst *et al*, 2001). More worryingly perhaps, some ‘sites’ overtly discourage patients to use conventional cancer therapies. No ‘website’ warns cancer patients about ACCs that have been demonstrated to be ineffective. Some of the recommended treatments are not curative but palliative or supportive by nature, e.g. aromatherapy, music therapy, massage, and this approach is undoubtedly more promising (Ernst, 2001).

How could ‘web sites’ be rendered safer and more informative for cancer patients? This is a most complex question, which needs careful consideration. We do not pretend to have all the answers. One step in the right direction might be to institute some sort of professional peer-review, which gives a ‘seal of approval’ to those ‘sites’ that pass the review. This would enable lay people to identify ‘sites’, which have been tested for quality. The HON code is an attempt of such a qualifier. Only 5 of 13 ‘sites’ analysed had this seal of approval; it is noteworthy that, by and large, these were the ‘sites’ that achieved a better rating than the rest, yet one of them presented a potential risk to cancer patients. The present system therefore has the potential to put patients at risk.

We conclude that an abundance of ‘web sites’ offer ACCs to cancer patients. The reliability of the advice thus provided is often poor. In order to avoid harm to our patients, ways of improving this situation should be found.
